# NOS3 Polymorphisms Can Influence the Effect of Multicomponent Training on Blood Pressure, Nitrite Concentration and Physical Fitness in Prehypertensive and Hypertensive Older Adult Women

**DOI:** 10.3389/fphys.2021.566023

**Published:** 2021-03-10

**Authors:** Átila Alexandre Trapé, Jhennyfer Aline Lima Rodrigues, Letícia Perticarrara Ferezin, Gustavo Duarte Ferrari, Elisangela Aparecida da Silva Lizzi, Vitor Nolasco de Moraes, Roberta Fernanda da Silva, Anderson Saranz Zago, Javier Brazo-Sayavera, Carlos Roberto Bueno Júnior

**Affiliations:** ^1^Ribeirão Preto College of Nursing, University of São Paulo (USP), Ribeirão Preto, Brazil; ^2^School of Physical Education and Sport of Ribeirão Preto, USP, Ribeirão Preto, Brazil; ^3^Ribeirão Preto Medical School, USP, Ribeirão Preto, Brazil; ^4^Academic Department of Mathematics, Federal University of Technology – Paraná, Cornélio Procópio, Brazil; ^5^Department of Physical Education, São Paulo State University, Bauru, Brazil; ^6^Polo de Desarrollo Universitario EFISAL, Centro Universitario Regional Noreste, Universidad de la República (UDELAR), Rivera, Uruguay

**Keywords:** aging, exercise training, genotype, nitric oxide, physical activity

## Abstract

Associations of endothelial nitric oxide synthase (NOS3) polymorphisms with hypertension and response to exercise training in prehypertensive and hypertensive older adult women remain unclear. This study used a multicomponent program (various capacities and motor skills) in the physical training intervention. It analyzed the influence of NOS3 polymorphisms [−786T > C, 894G > T (Glu298Asp), and intron 4b/a] on the response of blood pressure (BP), nitrite concentration, and physical fitness in older adult women. Fifty-two participants aged between 50 and 80 underwent body mass index, BP, 6-min walk, elbow flexion, and sit and stand-up tests to assess physical fitness. The intervention duration was 12 weeks, twice a week, on non-consecutive days. Each session lasted 90 min, maintaining an intensity between 13 (moderate) and 15 (intense), controlled by the Subjective Effort Perception Scale. Plasma/blood samples were collected to assess nitrite concentration and genotyping. The statistical analysis included Fisher’s exact test and linear mixed-effects models. The multicomponent training’s positive effect was observed with a similar response in both prehypertensive and hypertensive groups. However, carriers of different genotypes demonstrated different responses to training: the decreases in systolic and diastolic BP and increases in nitrite expected from the physical training were smaller in variant genotype than ancestral genotype carriers, especially in the hypertensive group. At positions −786T > C and Glu298Asp, only the ancestral genotypes showed a decrease in diastolic BP (Δ% = −8.1, and Δ% = −6.5, respectively) and an increase on nitrite (Δ% = 19.1, and Δ% = 24.1, respectively) in the hypertensive group. Our results show that the benefits of a multicomponent training intervention seem to be genotype-dependent. It should be possible to consider genetic variants when selecting an exercise treatment intervention.

## Introduction

Population aging is a worldwide phenomenon, being a reason to celebrate and a challenge (UNFPA, 2012). Human aging is defined as a dynamic and progressive process in which there are morphological, functional, biochemical, and psychological alterations causing a lower capacity of the biological system to maintain and repair itself (Rattan, 2014). Among these changes, it is worth noting the decrease in physical fitness (Chodzko-Zajko et al., 2009) and greater vulnerability to chronic diseases, especially cardiovascular diseases (North and Sinclair, 2012; Malachias et al., 2016), highlighting hypertension among the modifiable risk factors (Malachias et al., 2016).

The etiology of hypertension is multifactorial and may involve genetic, environmental, and psychological aspects. Neural and humoral factors are considered a mechanism to control blood pressure (BP), and some alterations could result in higher or lower levels of BP (Parati et al., 2013). Among these alterations in the humoral factors, one important BP control mechanism is the nitric oxide (NO) concentration. The reduction in NO production may be related to aging, a sedentary lifestyle, and genetic polymorphisms. Reduced NO production is directly associated with impairment in vasodilation, which causes an increase in peripheral vascular resistance and raises BP values; reduced NO production is associated with increased BP values, especially during the aging process (Vanhoutte, 2003; Rush et al., 2005; Moncada and Higgs, 2006; Trape et al., 2013; Jacomini et al., 2016).

It is known that endothelial cells are responsible for synthesis, metabolism, and the release of a wide variety of mediators that regulate vascular tone, with NO of paramount importance due to its role in BP control. NO can be produced in the endothelial cells by NO synthase (NOS3) and is responsive to physical exercise due to shear stress in vessel walls (Moncada and Higgs, 2006; da Silva et al., 2014; Jacomini et al., 2016). Therefore, the benefit of physical fitness is that it may increase NO concentration and decrease BP. However, improvement in BP values is still controversial, and different behavior can be found among individuals (Sponton et al., 2010, 2014; Zago et al., 2010; Esposti et al., 2011; da Silva et al., 2014). Although lifestyles with effective physical training and nutrition interventions can influence BP levels, the genetic influence has received special attention. Therefore, it is crucial to investigate the relationships and interactions between genetic polymorphisms, aging, and lifestyle changes, such as the response to physical exercise in prehypertensive and hypertensive people (Bouchard et al., 2015; Karasik and Newman, 2015).

Regarding the health issues that accompany aging, physical exercise regular practice can be considered one of the main actions that counteract detrimental changes. Thus, a multicomponent exercise protocol may be appropriate (Neves et al., 2016) due to the various motor skills and capacities affected by the aging process. This type of physical exercise program is following the official position of the American College of Sports Medicine (ACSM) (Chodzko-Zajko et al., 2009; Garber et al., 2011), which includes the training of various capacities and motor skills (aerobic capacity, muscular strength, flexibility, coordination, agility, and balance).

Among the many existing polymorphisms, genetic variants in the gene encoding NOS3 [−786T > C, 894G > T (Glu298Asp), and intron 4] may potentially explain the difference in the magnitude of changes in the aging process (Karasik and Newman, 2015). This study can explain why some people present more physical training benefits than others (Bouchard et al., 2015). In this way, the association between NOS3 polymorphisms and hypertension has been demonstrated in some studies (Casas et al., 2006; Zintzaras et al., 2006; Pereira et al., 2007). However, the associations of NOS3 polymorphisms with hypertension and the response to multicomponent physical training remain unclear. Similar previous studies found an influence of the genetic polymorphisms on the effect of physical training and others no. It is essential to highlight that all of them have been performed using only aerobic exercise as intervention (Sponton et al., 2010, 2014; Zago et al., 2010; Esposti et al., 2011; Rezende et al., 2011). Due to the lack of information on different protocols, it seems interesting to explore other proposals as the above-mentioned multicomponent training (various capacities and motor skills), which could be adequate for global analysis of the aging changes process. Some information has been published about NOS3 haplotype analysis in the effect of multicomponent training in older adult women with different health conditions in the same group (Trape et al., 2017). Therefore, it would be interesting to compare health parameter responses to multicomponent training in prehypertensive and hypertensive groups in a NOS3 genotype analysis.

The purpose of this study was to investigate the influence of NOS3 polymorphisms [−786T > C, 894G > T (Glu298Asp) and intron 4b/a] on the response of BP, nitrite concentration, and physical fitness of older adults, prehypertensive and hypertensive women, who underwent a multicomponent training intervention. The present study hypothesized that the prehypertensive and hypertensive groups would demonstrate different responses to physical training; and the carriers of the “C” allele of the −786 T > C polymorphism, the “a” allele of the intron 4b/a polymorphism, and GluAsp or AspAsp genotypes of the gene 894G > T (Glu298Asp) polymorphism (variant genotypes) would be less responsive to physical training.

## Materials and Methods

### Participants and Study Design

**FIGURE 1:**
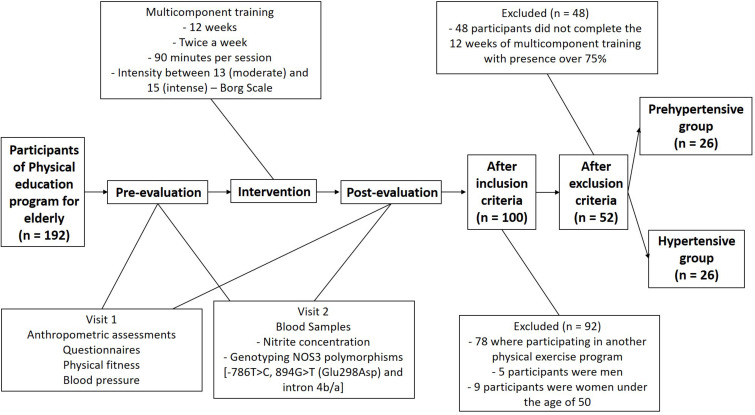
Flow chart – study design and sample selection.

The Ethics Committee approved this research project of the Faculty of Philosophy, Sciences, and Letters of Ribeirão Preto, University of São Paulo (24579513.4.0000.5407). The sample size was established by convenience based on the participants from an extension project. Participants from the extension project of a Physical Education program for the elderly at the School of Physical Education and Sport of Ribeirão Preto (University of São Paulo) were invited to participate in this study. Each month a group realized the assessments and began training. After the 12 weeks, the reevaluation occurred, and at the same time, a new group performed the assessments and started the training. It is important to note that participants continued in the extension project even after the study 12-week period. The research team allocated participants to the groups based on their BP: prehypertensive and hypertensive. The flow of participants and study design is illustrated in Figure 1. Before starting the project’s participation, a group meeting was organized to inform the participants about the study protocol. All participants signed a free and informed consent form after all their questions had been answered. Medical records of the participants were obtained. The following inclusion criteria were considered: no participation in any other physical exercise program 6 months before and during the intervention, being female and aged between 50 and 80. Exclusion criteria consisted of: any medical condition or musculoskeletal problems that could prevent the performance of the motor tests and physical training program; a body mass index (BMI) >35 kg/m^2^; maximal systolic BP > 160 mmHg and maximal diastolic BP > 100 mmHg; and presence below 75% in the activities proposed by the intervention.

### Intervention and Evaluation

The intervention duration was 12 weeks, twice a week, on non-consecutive days, and each session lasted 90 min. The sessions were divided into four parts: (1) Warm-up, including walking, exercise on a bicycle, dynamic stretching exercises, coordination, balance, different movements using arms and legs, and some group games. These activities were always used with low intensity to prepare for the main part (about 20 to 30 min); (2) First, participants learned about the technique to squat, push up, jump and posture to do different exercises and participate in the activities. Strength and agility exercises were performed in the form of a circuit by time using elastics, balls, sticks, free weights, and body weight. Participants had from six to eight activities to train for 1.5 min and 1 min to rest and change the activity (about 30 to 40 min); (3) Aerobic (walking, running, and exercise on a bicycle) and ludic activities (dances with a teacher coordinating or group games with movements) (about 20 to 30 min); and (4) “Back to calm,” relaxation, massage, breathing and stretching exercises (about 10 min). The intensity of the training was controlled by the Subjective Effort Perception Scale (Borg, 1970), maintaining an intensity of between 13 (moderate) and 15 (intense). Assessments were performed before and after 12 weeks of intervention. All participants carried out the same exercise protocol and the same assessments by the researchers blinded to the participants’ condition.

A questionnaire with open and closed questions was applied regarding hypertension based on the medical diagnosis of sustained elevation of systolic BP equal to or greater than 140 and diastolic BP by 90 mmHg (“no,” “yes and without the use of medications,” and “yes with the use of medications”); general health condition (disease or risk factor for any specific disease); physical limitation for day-to-day activities or that could impede motor testing; and age. Body mass and height assessments were performed using a scale (graduation of 50 g) with a stadiometer (precision of 1.0 mm) (Welmy W200ALCD), enabling calculation of the BMI using the equation weight/height^2^. BP was assessed using an automatic arm digital pressure gage (OMRON brand, model HEM-7113, Japan) following the Brazilian Guidelines for Hypertension VII recommendations. Blood pressure was measured three times, and when a measure with a difference higher than 10 mmHg for SBP or 5 mmHg for DBP was found, a fourth measure was done. The participants classified as prehypertensive did not use medications to BP and demonstrated systolic BP between 121 and 139 and/or diastolic BP between 81 and 89 mmHg (Malachias et al., 2016).

Aerobic capacity was assessed using the 6-min walk test (distance traveled on a rectangular route measuring 4.57 m × 18.28 m—the participant was required to walk as fast as possible, without running); muscular endurance of the upper limbs was assessed by the elbow extension and flexion test (highest number of complete repetitions of extension and elbow flexion with the dominant arm in 30 s with the participant sitting and holding a dumbbell of 2.27 kg); and the lower limbs were assessed using the sit and stand up test (highest number of complete repetitions in 30 s to sit and stand up from a chair—the participant was required to keep their arms crossed at the front of the trunk and touch the chair with the gluteus in each movement) (Rikli and Jones, 2008).

Besides, two questionnaires were applied: International Physical Activity Questionnaire (IPAQ) (Matsudo et al., 2001) and Food Consumption Marker Form (indicates the frequency of consumption of 10 food groups in the days of a regular week, such as raw salad, cooked vegetables, beans, fruits, milk, sweet and salty snacks, among others) (Brasil, 2008). Although not included in the objectives, these two instruments were used to control for confounding factors, and the participants were oriented to maintain the same eating and physical activity habits during the 12-week training period.

### Blood Analysis

Blood samples were drawn in the morning (7:00–8:30 a.m.) after 12-h overnight fasting from the antecubital vein and stored in EDTA tubes. Participants had a rest day and were instructed to avoid foods with a high concentration of nitrate daily before collection, such as beetroot, arugula, spinach, lettuce, and others. Plasma was separated by 2,000 g centrifugation for 4 min at 24°C and was used for the nitrite concentration analysis. Whole blood was used for genotyping.

### Nitrite (NO_2_) Concentration

For the NO analysis, an indirect determination method was used through the nitrite (NO_2_) plasma, the product of NO’s reaction with O_2_. Plasma aliquots were analyzed in duplicate for their nitrite content using ozone-based chemiluminescence (Sievers Model 280 NO Analyzer, Sievers, United States). The data were analyzed using the Origin Lab 6.1 program (Pinheiro et al., 2012).

### Genotyping

The method of DNA extraction used was salting out (Lahiri and Nurnberger, 1991). The purity and DNA concentration of the sample was assessed by spectrophotometry (BioDrop μlite PC). The ratios 260/230 and 280/260 were assessed, and the level of purity adopted as a satisfactory minimum was 1.7 for each of the rates.

The NOS3 single nucleotide polymorphism (SNP) at the position −786T > C (rs2070744) was determined by real-time PCR (StepOnePlus equipment, Applied Biosystems, United States). The reaction was carried out using Custom Taqman allele discrimination assay (re-synthesis part number AH5I790, Thermo Fisher, United States) and JumpStart Ready Mix for Quantitative PCR (Sigma-Aldrich, United States). Preparation of the reactions was performed according to the manufacturer’s specifications (Vasconcellos et al., 2010). The NOS3 SNP at the position 894G > T (Glu298Asp) (rs1799983) was analyzed by conventional PCR followed by electrophoresis, using the following primers—forward: 5′-CATGAGGCTCAG CCCCAGAAC-3′; reverse: 5′-AGTCAATCCCTTTGG TGC TCAC-3′. The amplicon was digested overnight at 37°C using 2 U *Mbo*I enzyme followed by electrophoresis for 3 h on 2.5% agarose gel. The G allele yielded a fragment of 248 bp, and the T allele yielded fragments of 190 and 58 bp (Zago et al., 2010). Genotypes for the variable number of tandem repeats (VNTR) polymorphism in intron 4 were determined by conventional PCR, using the primers: forward 5′-AGG CCC TAT GGT AGT GCC TTT-3′ and reverse 5′-TCT CTT AGT GCT GTG GTC AC-3′ and fragment separation by electrophoresis for 3 h in 8% polyacrylamide gels. Fragments of 393 and 420 bp corresponded to the NOS3 alleles 4a and 4b, respectively (Marroni et al., 2005).

### Statistical Analysis

Data analysis was performed using Fisher’s exact test to verify the statistical association of categorical variables with time (food intake) and linear mixed-effects models (random and fixed effects) adjusted for age, food intake, and physical activity level, socioeconomic status, and body weight. The classes of linear mixed-effects models are extensions of linear regression models for data collected and summarized in groups. They are used to analyze data in which the answers are grouped (repeated measures for the same individual), as we have information for the same individual in the pre and post time, and the assumption of independence between observations in the same group is not adequate. This model assumes that the residue obtained by means of the difference between the values predicted by the model and the observed values has a normal distribution with mean 0 and constant variance (Schall, 1991). The effect size was used as a quantitative measure of the experimental effect’s magnitude (baseline versus post). Cutoff points are: *d* = 0.2 is a “small” effect size, *d* = 0.5 represents a “’medium” effect size, and 0.8 or more a “large” effect size (Cohen, 1988). In these analyses, a level of significance of 5% was considered, and the analysis was performed in SAS software (version 9.2) using the PROC MIXED and PROC TABULATE. The graphs were constructed using R software (version 3.2).

## Results

### General Data

Regarding the weekly frequency of intake of various foods, related to the frequency of consumption of 10 food groups in the days of a regular week, it was verified that there were no differences at baseline and post the multicomponent physical training intervention, showing that food was similar during the study protocol (Supplementary Data, Tables A and B). We observed an increase in moderate and vigorous physical activity related to participation in the physical activity program offered by this research regarding physical activity habits. The walking and sitting times are similar during the intervention (Supplementary Data, Table C). The results are similar when we observed the different groups, prehypertensive and hypertensive, and according to genotypes distribution.

In the same way, the percentage of individuals using anti-hypertensive drugs in the hypertensive group was 92.3%, and the distribution was homogeneous regardless of the genotypes [−786T > C: TT = 92.3%, TC + CC = 92.3%; Glu298Asp: GluGlu = 93.8%, GluAsp + AspAsp = 90.0%; intron 4 b/a: 4b4b = 92.3%, 4b4a + 4a4a = 90.9%]. There was no change in the use of medications between baseline and after the intervention.

The distribution of participant genotypes across the groups indicates that no participant was classified as 4a4a for intron 4b/a in either group, prehypertensive, or hypertensive (Table 1). The three polymorphisms’ distribution of genotypes showed no deviation from the Hardy-Weinberg equilibrium (*p* > 0.05) with allele frequencies at position −786T > C of 0.71 and 0.29 for T and C allele, respectively, in the prehypertensive group and 0.69 and 0.31 in the hypertensive group. The allele frequencies for G and T at position 894G > T (Glu298Asp) in the prehypertensive group were 0.75 and 0.25, respectively, and 0.79 and 0.21 in the hypertensive group. In intron 4b/a, the frequencies were 0.88 and 0.12 for 4b and 4a in the prehypertensive group and 0.79 and 0.21 in the hypertensive group.

**TABLE 1:** Participants’ genotypes distribution.

	Prehypertensive		Hypertensive	
		
Genotypes	(*n* = 26)	%	(*n* = 26)	%
**−786T > C**				
TT	12	46.2	13	50.0
TC	13	50.0	10	38.5
CC	1	3.8	3	11.5
**Glu298Asp**				
GluGlu	14	53.9	16	61.6
GluAsp	11	42.3	9	34.6
AspAsp	1	3.8	1	3.8
**Intron 4**				
4b4b	20	76.9	15	57.7
4b4a	6	23.1	11	42.3
4a4a	0	0	0	0

Multicomponent training for 12 weeks decreased body weight [prehypertensive group: 70.7 (11.0); 69.1 (10.6) kg; *p* < 0.05—hypertensive group: 71.5 (11.4); 69.7 (11.3) kg; *p* < 0.05] and BMI [prehypertensive group: 28.1 (5.4); 27.4 (5.1) kg/m^2^; *p* < 0.05—hypertensive group: 28.8 (3.7); 28.1 (3.6) kg/m^2^; *p* < 0.05]. Multicomponent training was effective in promoting a reduction in systolic and diastolic BP, as well as an increase in nitrite concentration and the flexion elbow test, sit and stand up test, and 6-min walk test, in the prehypertensive and hypertensive groups (Table 2).

**TABLE 2:** Effects of multicomponent training on blood pressure, nitrite concentration, and physical fitness in 26 prehypertensive and 26 hypertensive older adult women.

	Prehypertensive (26)				Hypertensive (26)			
		
	Baseline	Post	ES	Δ%	Baseline	Post	ES	Δ%
Age (years)	61.7 (8.3)				62.2 (7.9)			
SBP (mmHg)	124.1 (8.6)	117.0(9.4)*	0.79	–5.7	140.9(14.7)^#^	131.2(15.3)*	0.64	–6.9
DBP (mmHg)	80.7 (9.5)	75.2(7)*	0.65	–6.8	85.0(8.7)^#^	80.3(9.2)*	0.52	–5.5
NO_2_ (nM)	105.3 (50)	142.5(78)*	0.57	35.5	118.7 (59.6)	140.5(61.3)*	0.36	18.7
EFT (reps)	16.9 (3.7)	20.0(4.3)*	0.77	18.3	17.3 (2.9)	20.2(3.9)*	0.84	16.8
SS (reps)	14.8 (3.7)	18.6(5.1)*	0,85	25.7	14.1 (3.0)	17.0(4.4)*	0.77	20.6
6 min WT (m)	525.2 (65.6)	563.6(64)*	0.59	7.3	498.5 (57.6)	555.3(56.1)*	0.99	11.4

Data are reported as means (SD) baseline and post 12 weeks of multicomponent training for 52 women. ES, effect size; SBP, systolic blood pressure; DBP, diastolic blood pressure; NO_2_, nitrite concentration; EFT, elbow flexion test; SS, sit and stand up test; 6 min WT, 6-min walk test. *p < 0.05 compared with baseline (the same group), ^#^*p* < 0.05 hypertensive compared with prehypertensive group baseline. Linear mixed effects models.

### Genotype Analysis

In the NOS3 genotype analysis, besides the division into prehypertensive and hypertensive individuals, participants were divided into ancestral genotype carriers [Prehypertensive: −786T > C – TT (*n* = 12), Glu298Asp: GluGlu (*n* = 14), intron 4b/a: 4b4b (*n* = 20); Hypertensive: −786T > C – TT (*n* = 13), Glu298Asp: GluGlu (*n* = 16), intron 4b/a: 4b4b (*n* = 15)] and variant genotype carriers [Prehypertensive: −786T > C – TC + CC (*n* = 14), Glu298Asp: GluAsp + AspAsp (*n* = 12), intron 4: 4b4a + 4a4a (*n* = 6); Hypertensive: −786T > C – TC + CC (*n* = 13), Glu298Asp: GluAsp + AspAsp (*n* = 10), intron 4: 4b4a + 4a4a (*n* = 11)].

**FIGURE 2:**
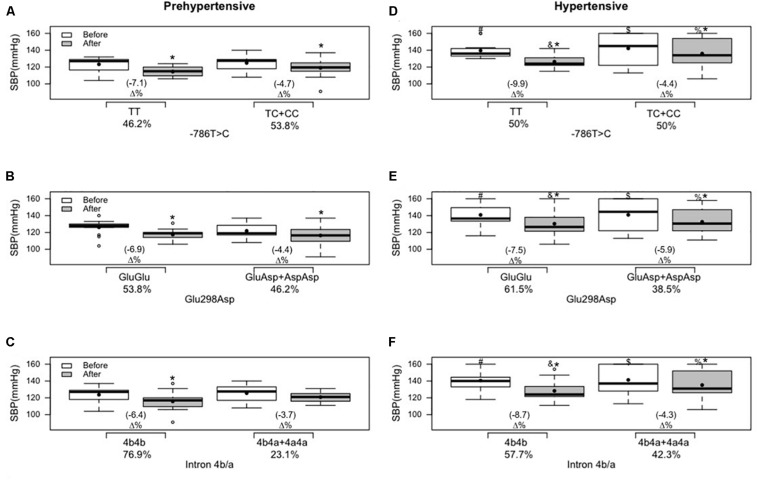
Effects of 12 weeks of multicomponent training on systolic blood pressure (SBP) of 26 prehypertensive and 26 hypertensive older adult women with or without variant genotypes for the NOS3 gene at positions –786T > C, 894G > T (Glu298Asp), and intron 4b/a. *p < 0.05 compared with before the intervention (the same group). ^#^*p* < 0.05 ancestral genotype of the hypertensive group compared with the ancestral genotype of the prehypertensive group (“TT” versus “TT”; “GluGlu” versus “GluGlu”; “4b4b” versus “4b4b”) before the intervention. $*p* < 0.05 variant genotypes of the hypertensive group compared with the variant genotypes of the prehypertensive group (“TC + CC” versus “TC + CC”; “GluAsp + AspAsp” versus “GluAsp + AspAsp”; “4b4a + 4a4a” versus “4b4a + 4a4a”) before the intervention. &*p* < 0.05 ancestral genotype of the hypertensive group compared with the ancestral genotype of the prehypertensive group (“TT” versus “TT”; “GluGlu” versus “GluGlu”; “4b4b” versus “4b4b”) after the intervention. %*p* < 0.05 variant genotypes of the hypertensive group compared with the variant genotypes of the prehypertensive group (“TC + CC” versus “TC + CC”; “GluAsp + AspAsp” versus “GluAsp + AspAsp”; “4b4a + 4a4a” versus “4b4a + 4a4a”) after the intervention. **(A)** Genotypes for the NOS3 gene at position −786T > C in the prehypertensive group. **(B)** Genotypes for the NOS3 gene at position 894G > T (Glu298Asp) in the prehypertensive group. **(C)** genotypes for the NOS3 gene at intron 4b/a in the prehypertensive group. **(D)** Genotypes for the NOS3 gene at position −786T > C in the hypertensive group. **(E)** Genotypes for the NOS3 gene at position 894G > T (Glu298Asp) in the hypertensive group. **(F)** Genotypes for the NOS3 gene at intron 4b/a in the hypertensive group. Linear mixed-effects models.

**FIGURE 3:**
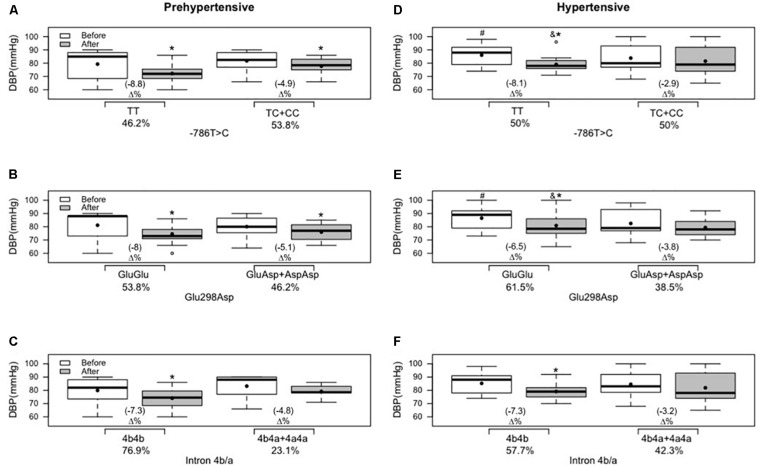
Effects of 12 weeks of multicomponent training on diastolic blood pressure (DBP) of 26 prehypertensive and 26 hypertensive older adult women with or without variant genotypes for the NOS3 gene at positions –786T > C, 894G > T (Glu298Asp), and intron 4b/a. *p < 0.05 compared with before the intervention (the same group). ^#^*p* < 0.05 ancestral genotype of the hypertensive group compared with the ancestral genotype of the prehypertensive group (“TT” versus “TT”; “GluGlu” versus “GluGlu”) before the intervention. &*p* < 0.05 ancestral genotype of the hypertensive group compared with the ancestral genotype of the prehypertensive group (“TT“versus “TT”; “GluGlu” versus “GluGlu”) after the intervention. **(A)** Genotypes for the NOS3 gene at position −786T > C in the prehypertensive group. **(B)** Genotypes for the NOS3 gene at position 894G > T (Glu298Asp) in the prehypertensive group. **(C)** genotypes for the NOS3 gene at intron 4b/a in the prehypertensive group. **(D)** Genotypes for the NOS3 gene at position −786T > C in the hypertensive group. **(E)** Genotypes for the NOS3 gene at position 894G > T (Glu298Asp) in the hypertensive group. **(F)** Genotypes for the NOS3 gene at intron 4b/a in the hypertensive group. Linear mixed-effects models.

In the cross-sectional analyses, systolic BP differed between hypertensive and prehypertensive groups under basal conditions and after the intervention, for the three genetic variants of NO3, in the comparison of ancestral and variant genotypes with the same group (Figure 2). Regarding diastolic BP, only ancestral genotype carriers in the hypertensive group at position −786T > C and Glu298Asp differed from the same variant genotypes of the prehypertensive group under basal conditions and after the intervention (Figure 3).

Only the variant genotype carriers of intron 4b/a did not present a reduction in systolic BP in the prehypertensive group (Figure 2C). Multicomponent training supported a statistical and significant decrease in systolic BP in all other ancestral and variant genotypes in prehypertensive and hypertensive groups (Figure 2).

**FIGURE 4:**
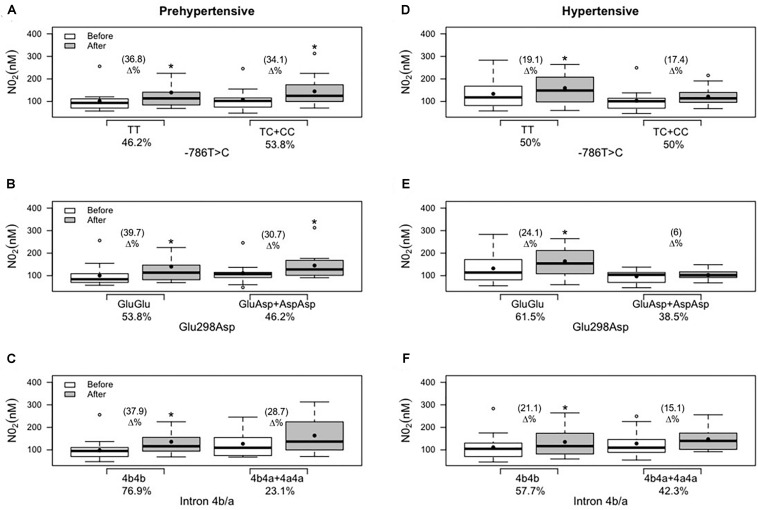
Effects of 12 weeks of multicomponent training on nitrite concentration (NO_2_) of 26 prehypertensive and 26 hypertensive older adult women with or without the variant genotypes for the NOS3 gene at positions –786T > C, 894G > T (Glu298Asp), and intron 4b/a. *p < 0.05 compared with the before intervention (the same group). **(A)** Genotypes for the NOS3 gene at position −786T > C in the prehypertensive group. **(B)** Genotypes for the NOS3 gene at position 894G > T (Glu298Asp) in the prehypertensive group. **(C)** Genotypes for the NOS3 gene at intron 4b/a in the prehypertensive group. **(D)** Genotypes for the NOS3 gene at position −786T > C in the hypertensive group. **(E)** Genotypes for the NOS3 gene at position 894G > T (Glu298Asp) in the hypertensive group. **(F)** Genotypes for the NOS3 gene at intron 4b/a in the hypertensive group. Linear mixed-effects models.

Regarding diastolic BP and nitrite concentration, in the prehypertensive group, except for variant genotype carriers of intron 4b/a (Figures 3C, 4C, respectively), a reduction in diastolic BP and an improvement in nitrite concentration were demonstrated in all other ancestral and variant genotypes (Figures 3, 4). In the hypertensive group, only the ancestral genotype carriers presented a statistical and significant decrease in diastolic BP (Figures 3D–F) and improved nitrite concentration (Figures 4D–F).

Regarding age, it was verified that there were no differences between the values of prehypertensive and hypertensive groups and between the ancestral genotype and variant genotype subgroups before the multicomponent physical training intervention. All groups demonstrated improvements in a similar fashion in physical fitness tests, except for variant genotype carriers at position −786T > C “TC + CC” that showed no improvement in the sit and stand up test in the hypertensive group, and intron 4b/a “4b4a + 4a4a” that did not present improvement in the elbow flexion test or the 6-min walk test in the prehypertensive group. In general, the multicomponent physical program effectively improved physical fitness in almost all genotype groups of the present study (Table 3).

**TABLE 3:** Effects of 12 weeks of multicomponent training on physical fitness of 26 prehypertensive and 26 hypertensive older adult women with or without variant genotypes for the NOS3 gene at position −786T > C, 894G > T (Glu298Asp) and intron 4b/a.

	Prehypertensive								Hypertensive							
		
	TT (12)				TC + CC (14)				TT (13)				TC + CC (13)			
										
	Baseline	Post	ES	Δ%	Baseline	Post	ES	Δ%	Baseline	Post	ES	Δ%	Baseline	Post	ES	Δ%
SBP (mmHg)	123.3 (8.5)	114.6 (6.0)	1.18	–7.1	124.9 (9.0)	119.0 (11.3)	0.58	–4.7	139.5 (9.9)	125.7 (7.4)	1.58	–9.9	142.2 (18.8)	136.0 (19.5)	0.32	–4.4
DBP (mmHg)	79.3 (11.4)	72.3 (6.6)	0.75	–8.8	81.8 (7.8)	77.8 (6.4)	0.56	–4.9	86.1 (7.4)	79.1 (6.4)	1.01	–8.1	83.9 (10.1)	81.5 (11.5)	0.22	–2.9
NO_2_ (nM)	102.1 (53.1)	139.7 (95.0)	0.48	36.8	108.0 (49.1)	144.8 (63.7)	0.65	34.1	133.8 (64.9)	159.4 (71.9)	0.37	19.1	103.6 (52.0)	121.6 (43.3)	0.38	17.4
EFT (reps)	16.1 (3.2)	19.0(4.1)*	0.79	18.0	17.6 (4.0)	20.9(4.4)*	0.78	18.8	16.9 (3.0)	20.1(3.1)*	1.04	18.9	17.7 (2.7)	20.2(4.8)*	0.64	14.1
SS (reps)	15.1 (4.7)	18.8(6.1)*	0.68	24.5	14.6 (2.7)	18.4(4.4)*	1.04	26.0	13.1 (1.6)	17.4(4.5)*	1.27	32.8	15.1 (3.7)	16.5 (4.3)	0.35	9.3
6 min WT (m)	531.0 (78.8)	573.5(83)*	0.53	8.0	520.3 (54.5)	555.1(43.3)*	0.71	6.7	485.9 (56.1)	544.9(66.0)*	1.78	12.1	511.0 (58.6)	565.8(44.2)*	1.06	10.7

	**GluGlu (14)**				**GluAsp + AspAsp (12)**				**GluGlu (14)**				**GluAsp + AspAsp (12)**			
										
	**Baseline**	**Post**		**Δ%**	**Baseline**	**Post**	**ES**	**Δ%**	**Baseline**	**Post**	**ES**	**Δ%**	**Baseline**	**Post**	**ES**	**Δ%**

SBP (mmHg)	126.1 (8.8)	117.4 (6.8)	1.10	–6.9	121.8 (8.2)	116.4 (12.0)	0.53	–4.4	140.8 (12.4)	130.3 (15.1)	0.76	–7.5	140.9 (18.7)	132.6 (16.2)	0.47	–5.9
DBP (mmHg)	81.1 (11.0)	74.6 (7.3)	0.69	–8.0	80.1 (7.9)	76.0 (6.8)	0.56	–5.1	86.5 (7.6)	80.9 (10.1)	0.63	–6.5	82.5 (10.2)	79.4 (7.9)	0.34	–3.8
NO_2_ (nM)	100.4 (52.2)	140.3 (92.6)	0.53	39.7	110.9 (48.9)	145.0 (60.8)	0.62	30.7	132.2 (69.9)	164.0 (66.2)	0.47	24.1	97.0 (29.7)	102.8 (23.9)	0.22	6.0
EFT (reps)	17.4 (3.3)	20.2(4.8)*	0.68	16.1	16.3 (4.1)	19.8(3.9)*	0.87	21.5	17.5 (3.0)	20.6(3.8)*	0.91	17.7	16.9 (2.7)	19.5(4.2)*	0.74	15.4
SS (reps)	16.4 (3.9)	20.0(5.5)*	0.76	22.0	13.0 (2.6)	16.9(4.4)*	1.08	30.0	14.4 (3.1)	17.4(4.1)*	0.83	20.8	13.5 (2.9)	16.2(4.8)*	0.68	20.0
6 min WT (m)	536.9 (77.6)	574.0(78.8)*	0.47	6.9	511.6 (48.0)	551.6(41.0)*	0.90	7.8	496.5 (50.1)	551.9(55.5)*	1.05	11.2	501.6 (70.8)	560.9(59.5)*	0.91	12.0

	**4b4b (20)**				**4b4a + 4a4a (6)**				**4b4b (15)**				**4b4a + 4a4a (11)**			
										
	**Baseline**	**Post**		**Δ%**	**Baseline**	**Post**	**ES**	**Δ%**	**Baseline**	**Post**	**ES**	**Δ%**	**Baseline**	**Post**	**ES**	**Δ%**

SBP (mmHg)	123.7 (7.9)	115.8 (9.8)	0.89	–6.4	125.5 (11.4)	120.8 (7.0)	0.50	–3.7	140.5 (11.7)	128.3 (12.0)	1.03	–8.7	141.3 (18.8)	135.2 (18.7)	0.33	–4.3
DBP (mmHg)	79.9 (9.6)	74.1 (7.1)	0.69	–7.3	83.2 (9.7)	79.2 (5.1)	0.52	–4.8	85.3 (8.0)	79.1 (6.7)	0.84	–7.3	84.6 (10.0)	81.9 (12.0)	0.24	–3.2
NO_2_ (nM)	98.7 (44.2)	136.1 (75.5)	0.60	37.9	127.2 (65.9)	163.7 (89.8)	0.46	28.7	111.7 (59.5)	135.3 (67.5)	0.37	21.1	128.2 (61.3)	147.5 (54.0)	0.33	15.1
EFT (reps)	16.4 (3.6)	19.8(3.9)*	0.91	20.7	18.7 (3.8)	20.8 (5.7)	0.43	11.2	17.3 (2.2)	19.7(3.2)*	0.87	13.9	17.3 (3.7)	20.7(4.8)*	0.79	19.7
SS (reps)	14.5 (3.9)	18.5(5.4)*	0.85	27.6	16.0 (3.1)	18.8(4.6)*	0.71	17.5	13.1 (1.9)	16.6(4.4)*	1.03	26.7	15.4 (3.7)	17.5(4.4)*	0.52	13.6
6 min WT (m)	517.2 (69.8)	561.0(70.8)*	0.62	8.5	551.9 (43.8)	572.4 (36.6)	0.51	3.7	501.8 (61.8)	553.7(51.8)*	0.91	10.3	494.0 (53.9)	557.5(64.0)*	1.07	12.9

*Data are reported as means (SD) baseline and post 12 weeks of multicomponent training for 52 women.*

Participants are divided into prehypertensive and hypertensive groups. ES, effect size; EFT, elbow flexion test; SS, sit and stand up test; 6 min WT, 6-min walk test.

*p < 0.05 compared with baseline (the same group). Linear mixed-effects models.

## Discussion

### General Data

The aim was to study the influence of the mentioned NOS3 polymorphisms on different outcomes in prehypertensive and hypertensive participants who underwent a multicomponent training program. In general, there were differences between groups after the intervention in systolic and diastolic BP, as expected, considering the two analyses performed (one only comparing prehypertensive and hypertensive individuals, and the other taking into account the analyses of each polymorphism for prehypertensive and hypertensive groups).

High BP is a risk factor for disability and mortality from cardiovascular disease (Jafar et al., 2017). Its reduction and the prevention of hypertension are in the first instance preferable through lifestyle changes (Caldarone et al., 2017). The current study has adopted a multicomponent training method because of the different components integrated, in agreement with ACSM guidelines (Chodzko-Zajko et al., 2009; Garber et al., 2011) that support many benefits of this training modality on cardiovascular health (Moraes et al., 2012; Neves et al., 2016). A meta-analysis (Cornelissen and Smart, 2013) demonstrated a 2.1 mmHg decrease in systolic BP and 1.7 mmHg in diastolic BP in prehypertensive subjects after aerobic endurance training. Such reductions in chronic levels are often more discreet but very important clinically. The current study has observed a 6.9 mmHg decrease in systolic BP and 5.5 mmHg in diastolic BP in the prehypertensive group after 12 weeks of multicomponent training. Thus, this BP status is a keystone to enroll patients in this kind of training program to reach a relevant BP decrease.

It is known that hemodynamic, humoral, and neural changes are associated with hypertension. Pharmacological therapy affects these variables and helps to control BP (Aronow et al., 2011). In this sense, most participants (92.3%) in the hypertensive group used antihypertensive drugs, and it is observed a reduction of 9.7 mmHg and 4.7 mmHg in systolic BP and diastolic BP, respectively. In line with the present results is a meta-analysis performed by Cornelissen and Smart (2013), where hypertensive individuals showed a reduction of 8.3 mmHg in systolic BP and 5.2 mmHg in diastolic BP, but after aerobic endurance training. It is important to emphasize that, according to Chobanian et al. (2003) a 2 mmHg reduction may decrease the risk of myocardial infarction by about 6% and the risk of developing coronary artery disease by 4%.

Previous studies have demonstrated that physical exercise is a good stimulus for increasing training status and promoting an increase in nitrite concentration (Jacomini et al., 2016), as observed in the current study.

Physical fitness can be considered an attribute that people possess or can achieve and is related to the capacity to perform physical activity and daily life activities (Caspersen et al., 1985). Besides, the multicomponent training increased muscular and cardiovascular endurance and effectively reduced BMI and systolic and diastolic BP in both groups.

### Genotype Analysis

Our results showed that the benefits of a multicomponent training intervention seem to be genotype-dependent. Carriers of different genotypes demonstrated different responses to training: the decreases in systolic and diastolic BP and increases in nitrite expected from the physical training were smaller in variant genotype than ancestral genotype carriers, especially in the hypertensive group.

Regarding the data of the three most commonly studied NOS3 polymorphisms [−786T > C, 894G > T (Glu298Asp), and intron 4b/a], studies show that they are not yet conclusive (Sponton et al., 2010, 2014; Zago et al., 2010; Esposti et al., 2011; Rezende et al., 2011). In the current study, it is possible to observe a different response to the multicomponent training between prehypertensive and hypertensive groups. It was evidenced that the improvement and response are associated with the genotype: the presence of the three variant genotypes [−786T > C: TC + CC, 894G > T (Glu298Asp): GluAsp + AspAsp and intron 4b/a: 4b4a + 4a4a] negatively affected the decrease in diastolic BP and the increase in nitrite concentration responses, mainly in the hypertensive group. All groups demonstrated improvements in a similar fashion in the physical fitness tests, except for the “TC + CC” genotypes carriers at position −786T > C and “4b4a + 4a4a” for intron 4b/a.

Rezende et al. (2011) and Sponton et al. (2010) examined the influence of NOS3 gene polymorphisms at position 894G > T (Glu298Asp) and position—786T > C, on the effect of long-term aerobic exercise training on the arterial BP in postmenopausal women. In the first study, related to 894G > T (Glu298Asp), they found a significant decrease in systolic and diastolic BP and a significant improvement in nitrite concentration in both groups, ancestral and variant genotypes, in a similar fashion. In the second study, related to −786T > C, systolic and diastolic BP values were reduced, and nitrite concentration was increased in both groups after 6 months of aerobic exercise, but women with ancestral genotype (“TT”) were more responsive to lowering BP and increasing nitrite concentration compared with those with variant genotypes (“TC + CC”). In the same way, our findings demonstrated a reduction in systolic and diastolic BP and improvement in nitrite concentration; however, it was possible to observe that the response of diastolic BP and nitrite concentration was genotype-dependent after 12 weeks of multicomponent training in the hypertensive group: carriers of the ancestral genotype (“GluGlu” and “TT”) demonstrated, respectively, reductions of 6.5 and 8.1% in diastolic BP (Figures 3D,E), and improvements of 19.1 and 24.1% in nitrite concentration (Figures 3D,E) and the variant genotypes (“GluAsp + AspAsp” and “TC + CC”) did not demonstrate statistical reduction.

In a study with 118 prehypertensive older subjects that underwent 6 months of supervised aerobic exercise, the participants did not demonstrate a decrease in systolic and diastolic BP or an increase in nitrite concentration in variant genotypes of NOS3 polymorphisms at positions −T786T > C and 894G > T (Glu298Asp) in an isolated analysis (Zago et al., 2010). In the same way, in the current study, it is possible to observe that variant genotypes did not affect the effect of physical exercise in the response of diastolic BP (Figures 3D–F) and nitrite concentration (Figures 4D–F) in the hypertensive group. However, a significant effect of multicomponent training was demonstrated on diastolic BP and nitrite concentration in the variant genotypes of −T786T > C and 894G > T (Glu298Asp) isolated polymorphisms in the prehypertensive group (Figures 3A,B, 4A,B). A study (Esposti et al., 2011) that analyzed each NOS3 polymorphism separately found similar improvements after eight weeks of aerobic exercise training in systolic and diastolic BP in all groups [ancestral and variant genotypes of −786T > C, 894G > T (Glu298Asp), and intron 4b/a]. However, they did not find improvement in nitrite/nitrate concentration. In the present study, it is observed that individuals who carry “a” allele (“4b4a + 4a4a” genotypes) present the worst responses to multicomponent training for diastolic BP and nitrite concentration independent of the group (prehypertensive or hypertensive). However, hypertensive individuals that presented variant genotypes of intron b/a (“4b4a + 4a4a”) demonstrated decreased systolic BP after training, and the same result was not found in the prehypertensive group. Similarly, Sponton et al. (2014) evaluated the relationship between exercise training and NOS3 polymorphisms using 24-h ambulatory BP monitoring and nitrate/nitrite levels. Aerobic exercise training was effective in lowering office BP as well as ambulatory BP monitoring. The variant genotypes for intron 4b/a mitigated aerobic exercise training beneficial effects for systolic and diastolic BP but was not associated with nitrite/nitrate levels. This fact explains the importance of multicomponent training in mitigating hypertensive women’s variant genotypes’ negative influence.

Multicomponent training is a modality that trains the individual globally with the work of different motor capacities such as strength, balance, coordination, and aerobic capacity. It was then expected that the benefits of these different types of exercises would add up in this study.

−786T > C is an SNP, which culminates in a significant reduction in enzyme activity in the promoter region of the NOS3 gene. This functional polymorphism reduces the expression of NOS when the C allele is present. It may explain the association between the risk of HT and coronary heart disease. 894G > T (Glu298Asp) is another SNP located in the exon seven region of the NOS3 gene, associated with lower production of NO, leading to an impairment of BP control. Intron 4b/a is related to low plasma levels of nitrite/nitrate. Genotype 4a + 4a showed a reduction in 20% of these metabolites concentration, and it is associated with a higher risk of hypertension. Thereby, a lower level of physical activity is associated with a high prevalence of hypertension (Casas et al., 2006; Sponton et al., 2014). da Silva et al. (2014) demonstrated that even in individuals with risk alleles, maintaining an adequate training status could help keep normal BP values.

The different responses presented in the current study results and previous studies can be explained by differences in the sample (age, sex, and ethnicity), baseline blood pressure values, training interventions (aerobic and multicomponent training), and types of evaluations.

Although comparison of the magnitude of change between ancestral and variant genotypes was not in the design or purpose of this study, when ancestral and variant genotypes presented changes in BP, it is possible to observe, descriptively, trends toward a better response (**Δ%**) in decreasing systolic BP in the ancestral genotype (“TT” and “GluGlu”) compared to the variant genotypes (“TC + CC” and “GluAsp + AspAsp”) in the prehypertensive and hypertensive groups [Figures 2A,D at position −786T > C; Figures 2B,E at position 894G > T (Glu298Asp)]; with the same result for systolic BP for the ancestral genotype of intron 4b/a (“4b4b”) compared to the variant genotypes (“4b4a + 4a4a”) but only in the hypertensive group (Figure 2F); and, for diastolic BP in the prehypertensive group [Figure 3A at position −786T > C; Figure 3B at position 894G > T (Glu298Asp)].

A limitation to be pointed out are no information about menopause and the number of participants (*n* = 6) in the variant genotype carriers of intron 4b/a “4b4a + 4a4a” in the prehypertensive group. Despite the low number of data, considering a genotyping study, no prior information about the genetic variants was known. This result is consistent with other studies presented in the discussion. Besides, almost all the hypertensive individuals in the current study were under medication conditions regarding the use of drugs. During the multicomponent training intervention, there was no change regarding these habits. In this way, the current work demonstrated the complementary effect of multicomponent physical training on the BP of hypertensive individuals who use medication.

The present study’s novelty uses a multicomponent program in the physical training intervention instead of aerobic exercise. In addition to isolated analysis of prehypertensive and hypertensive response groups, analysis of each NOS3 polymorphism was performed (−786T > C, Glu298Asp and intron 4b/a) in these groups.

In conclusion, a 12-week multicomponent training in prehypertensive and hypertensive participants showed a reduction in BP and an increase in NO concentration. However, it is possible to observe that the response is associated with the genotype in both groups: variant genotypes presented a lower decrease in systolic and diastolic BP and a lower increase in nitrite concentration than the ancestral genotypes, mainly in the hypertensive group. This knowledge of genotype differences in responsiveness to exercise training could make it feasible in the future, in which exercise program can lead to more health benefits for each individual. In this way, an exercise program should be an essential part of preventative and therapeutic strategies.

## Data Availability Statement

The raw data supporting the conclusions of this article will be made available by the authors, without undue reservation.

## Ethics Statement

The studies involving human participants were reviewed and approved by the Ethics Committee of the Faculty of Philosophy, Sciences, and Letters of Ribeirão Preto, University of São Paulo (CAAE 24579513.4.0000.5407). The patients/participants provided their written informed consent to participate in this study.

## Author Contributions

AT, AZ, EL, and CB contributed to the study design. AT, JR, LF, GF, and VM contributed to the recruitment of participants, evaluations, blood analysis, and implementation of the intervention. EL and JB-S contributed to the data analysis. AT, JR, and JB-S contributed to the manuscript drafting. All authors contributed to the review of the drafted manuscript.

## Conflict of Interest

The authors declare that the research was conducted in the absence of any commercial or financial relationships that could be construed as a potential conflict of interest.

## References

[B1] Aronow W. S. Fleg J. L. Pepine C. J. Artinian N. T. Bakris G. Brown A. S. (2011). ACCF/AHA 2011 expert consensus document on hypertension in the elderly: a report of the american college of cardiology foundation task force on clinical expert consensus documents developed in collaboration with the american academy of neurology, american geriatrics society, american society for preventive cardiology, american society of hypertension, american society of nephrology, association of black cardiologists, and european society of hypertension. *J. Am. Coll. Cardiol.* 57 2037–2114. 10.1016/j.jacc.2011.01.008 21524875

[B2] Borg G. (1970). Perceived exertion as an indicator of somatic stress. *Scand. J. Rehabil. Med.* 2 92–98. 5523831

[B3] Bouchard C. Antunes-Correa L. M. Ashley E. A. Franklin N. Hwang P. M. Mattsson C. M. (2015). Personalized preventive medicine: genetics and the response to regular exercise in preventive interventions. *Prog. Cardiovasc. Dis.* 57 337–346. 10.1016/j.pcad.2014.08.005 25559061 PMC4285566

[B4] Brasil (2008). *Protocolos do Sistema de Vigilância Alimentar e Nutricional-SISVAN na Assistência à Saúde.* Brasilia: Ministério da Saúde.

[B5] Caldarone E. Severi P. Lombardi M. D’Emidio S. Mazza A. Bendini M. G. (2017). Hypertensive response to exercise and exercise training in hypertension: odd couple no more. *J. Clin. Hypertens.* 23:11. 10.1186/s40885-017-0067-z 28588902 PMC5455108

[B6] Casas J. P. Cavalleri G. L. Bautista L. E. Smeeth L. Humphries S. E. Hingorani A. D. (2006). Endothelial nitric oxide synthase gene polymorphisms and cardiovascular disease: a HuGE review. *Am. J. Epidemiol.* 164 921–935. 10.1093/aje/kwj302 17018701

[B7] Caspersen C. J. Powell K. E. Christenson G. M. (1985). Physical activity, exercise, and physical fitness: definitions and distinctions for health-related research. *Public Health Rep.* 100 126–131. 3920711 PMC1424733

[B8] Chobanian A. V. Bakris G. L. Black H. R. Cushman W. C. Green L. A. Izzo J. L. Jr. (2003). The seventh report of the joint national committee on prevention, detection, evaluation, and treatment of high blood pressure: the JNC 7 report. *J. Am. Med. Assoc.* 289 2560–2572. 10.1001/jama.289.19.2560 12748199

[B9] Chodzko-Zajko W. J. Proctor D. N. Fiatarone Singh M. A. Minson C. T. Nigg C. R. Salem G. J. (2009). American college of sports medicine position stand. exercise and physical activity for older adults. *Med. Sci. Sports Exercise* 41 1510–1530. 10.1249/MSS.0b013e3181a0c95c 19516148

[B10] Cohen J. (ed.). (1988). “The concepts of power analysis,” in *Statistical Power Analysis for the Behavioral Sciences.* Chap. 1 (Hillsdale, NJ: Academic Press), 1–17.

[B11] Cornelissen V. A. Smart N. A. (2013). Exercise training for blood pressure: a systematic review and meta-analysis. *J. Am. Heart Assoc.* 2:e004473. 10.1161/JAHA.112.004473 23525435 PMC3603230

[B12] da Silva R. F. Sertorio J. T. Lacchini R. Trape A. A. Tanus-Santos J. E. Rush J. W. (2014). Influence of training status and eNOS haplotypes on plasma nitrite concentrations in normotensive older adults: a hypothesis-generating study. *Aging Clin. Exp. Res.* 26 591–598. 10.1007/s40520-014-0218-y 24760600

[B13] Esposti R. D. Sponton C. H. Malagrino P. A. Carvalho F. C. Peres E. Puga G. M. (2011). Influence of eNOS gene polymorphism on cardiometabolic parameters in response to physical training in postmenopausal women. *Braz. J. Med. Biol. Res.* 44 855–863. 10.1590/s0100-879x2011007500106 21956531

[B14] Garber C. E. Blissmer B. Deschenes M. R. Franklin B. A. Lamonte M. J. Lee I. M. (2011). American college of sports medicine position stand. quantity and quality of exercise for developing and maintaining cardiorespiratory, musculoskeletal, and neuromotor fitness in apparently healthy adults: guidance for prescribing exercise. *Med. Sci. Sports Exercise* 43 1334–1359. 10.1249/MSS.0b013e318213fefb 21694556

[B15] Jacomini A. M. de Souza H. C. Dias Dda S. Brito Jde O. Pinheiro L. C. da Silva A. B. (2016). Training status as a marker of the relationship between nitric oxide, oxidative stress, and blood pressure in older adult women. *Oxid. Med. Cell. Longev.* 2016:8262383. 10.1155/2016/8262383 26697141 PMC4678091

[B16] Jafar T. H. Jehan I. de Silva H. A. Naheed A. Gandhi M. Assam P. (2017). Multicomponent intervention versus usual care for management of hypertension in rural Bangladesh, Pakistan and Sri Lanka: study protocol for a cluster randomized controlled trial. *Trials* 18:272. 10.1186/s13063-017-2018-0 28606184 PMC5469065

[B17] Karasik D. Newman A. (2015). Models to explore genetics of human aging. *Adv. Exp. Med. Biol.* 847 141–161. 10.1007/978-1-4939-2404-2_7 25916590

[B18] Lahiri D. K. Nurnberger J. I. Jr. (1991). A rapid non-enzymatic method for the preparation of HMW DNA from blood for RFLP studies. *Nucleic Acids Res.* 19:5444. 10.1093/nar/19.19.5444 1681511 PMC328920

[B19] Malachias M. V. B. Franco R. J. Forjaz C. L. M. Pierin A. M. G. Gowdak M. M. Klein M. (2016). 7th Brazilian guideline of arterial hypertension. *Arq. Bras. Cardiol.* 107(3 Suppl. 3) 30–34. 10.5935/abc.20160156 27819385 PMC5319467

[B20] Marroni A. S. Metzger I. F. Souza-Costa D. C. Nagassaki S. Sandrim V. C. Correa R. X. (2005). Consistent interethnic differences in the distribution of clinically relevant endothelial nitric oxide synthase genetic polymorphisms. *Nitric Oxide* 12 177–182. 10.1016/j.niox.2005.02.002 15797845

[B21] Matsudo S. Araujo T. Matsudo V. Andrade D. Andrade E. Oliveira L. C. (2001). Questionário Internacional de Atividade Física (I-PAQ): estudo de validade e reprodutibilidade no Brasil. *Rev. Atividade Física Saúde* 6 5–18.

[B22] Moncada S. Higgs E. A. (2006). The discovery of nitric oxide and its role in vascular biology. *Br. J. Pharmacol.* 147(Suppl. 1) S193–S201. 10.1038/sj.bjp.0706458 16402104 PMC1760731

[B23] Moraes W. M. Souza P. R. Pinheiro M. H. Irigoyen M. C. Medeiros A. Koike M. K. (2012). Exercise training program based on minimum weekly frequencies: effects on blood pressure and physical fitness in elderly hypertensive patients. *Rev. Brasil. Fisioter.* 16 114–121. 10.1590/s1413-35552012005000013 22481693

[B24] Neves L. M. Diniz T. A. Rossi F. E. Fortaleza A. C. D. S. Horimoto E. T. Geraldo V. D. O. (2016). The effect of different training modalities on physical fitness in women over 50 years of age. *Motriz* 22 319–326. 10.1590/s1980-6574201600040016

[B25] North B. J. Sinclair D. A. (2012). The intersection between aging and cardiovascular disease. *Circ. Res.* 110 1097–1108. 10.1161/CIRCRESAHA.111.246876 22499900 PMC3366686

[B26] Parati G. Ochoa J. E. Lombardi C. Bilo G. (2013). Assessment and management of blood-pressure variability. *Nat. Rev. Cardiol.* 10 143–155. 10.1038/nrcardio.2013.1 23399972

[B27] Pereira T. V. Rudnicki M. Cheung B. M. Baum L. Yamada Y. Oliveira P. S. (2007). Three endothelial nitric oxide (NOS3) gene polymorphisms in hypertensive and normotensive individuals: meta-analysis of 53 studies reveals evidence of publication bias. *J. Hypertens.* 25 1763–1774. 10.1097/HJH.0b013e3281de740d 17762636

[B28] Pinheiro L. C. Montenegro M. F. Amaral J. H. Ferreira G. C. Oliveira A. M. Tanus-Santos J. E. (2012). Increase in gastric pH reduces hypotensive effect of oral sodium nitrite in rats. *Free Radic. Biol. Med.* 53 701–709. 10.1016/j.freeradbiomed.2012.06.001 22721923

[B29] Rattan S. I. (2014). Aging is not a disease: implications for intervention. *Aging Dis.* 5 196–202. 10.14336/AD.2014.0500196 24900942 PMC4037311

[B30] Rezende T. M. Sponton C. H. Malagrino P. A. Bezerra M. A. Penteado C. F. Zanesco A. (2011). Effect of exercise training on the cardiovascular and biochemical parameters in women with eNOS gene polymorphism. *Arch. Physiol. Biochem.* 117 265–269. 10.3109/13813455.2011.596548 21801125

[B31] Rikli R. E. Jones J. C. (2008). *Teste De Aptidão Física Para Idosos.* Barueri: Manole.

[B32] Rush J. W. Green H. J. Maclean D. A. Code L. M. (2005). Oxidative stress and nitric oxide synthase in skeletal muscles of rats with post-infarction, compensated chronic heart failure. *Acta Physiol. Scand.* 185 211–218. 10.1111/j.1365-201X.2005.01479.x 16218926

[B33] Schall R. (1991). Estimation in generalized linear models with random effects. *Biometrika* 78 719–727. 10.2307/2336923

[B34] Sponton C. H. Esposti R. Rodovalho C. M. Ferreira M. J. Jarrete A. P. Anaruma C. P. (2014). The presence of the NOS3 gene polymorphism for intron 4 mitigates the beneficial effects of exercise training on ambulatory blood pressure monitoring in adults. *Am. J. Physiol. Heart Circ. Physiol.* 306 H1679–H1691. 10.1152/ajpheart.00844.2013 24748593

[B35] Sponton C. H. Rezende T. M. Mallagrino P. A. Franco-Penteado C. F. Bezerra M. A. Zanesco A. (2010). Women with TT genotype for eNOS gene are more responsive in lowering blood pressure in response to exercise. *Eur. J. Cardiovasc. Prev. Rehabil.* 17 676–681. 10.1097/HJR.0b013e32833a1301 20436351

[B36] Trape A. A. Jacomini A. M. Muniz J. J. Sertorio J. T. Tanus-Santos J. E. do Amaral S. L. (2013). The relationship between training status, blood pressure and uric acid in adults and elderly. *BMC Cardiovasc. Disord.* 13:44. 10.1186/1471-2261-13-44 23799981 PMC3695764

[B37] Trape A. A. Lizzi E. Goncalves T. C. P. Rodrigues J. A. L. Tavares S. S. Lacchini R. (2017). Effect of multicomponent training on blood pressure, nitric oxide, redox status, and physical fitness in older adult women: influence of endothelial nitric oxide synthase (NOS3) haplotypes. *Oxid. Med. Cell. Longev.* 2017:2578950. 10.1155/2017/2578950 29104725 PMC5618760

[B38] UNFPA (2012). *Ageing in the Twenty-First Century: A Celebration and A Challenge.* New York, NY: United Nations Population Fund.

[B39] Vanhoutte P. M. (2003). Endothelial control of vasomotor function: from health to coronary disease. *Circulation* 67 572–575. 10.1253/circj.67.572 12845177

[B40] Vasconcellos V. Lacchini R. Jacob-Ferreira A. L. Sales M. L. Ferreira-Sae M. C. Schreiber R. (2010). Endothelial nitric oxide synthase haplotypes associated with hypertension do not predispose to cardiac hypertrophy. *DNA Cell Biol.* 29 171–176. 10.1089/dna.2009.0955 20070154

[B41] Zago A. S. Park J. Y. Fenty-Stewart N. Kokubun E. Brown M. D. (2010). Effects of aerobic exercise on the blood pressure, oxidative stress and eNOS gene polymorphism in prehypertensive older people. *Eur. J. Appl. Physiol.* 110 825–832. 10.1007/s00421-010-1568-6 20614130

[B42] Zintzaras E. Kitsios G. Stefanidis I. (2006). Endothelial NO synthase gene polymorphisms and hypertension: a meta-analysis. *Hypertension* 48 700–710. 10.1161/01.HYP.0000238124.91161.02 16940230

