# Supplementary material for: NOS3 Polymorphisms Can Influence the Effect of Multicomponent Training on Blood Pressure, Nitrite Concentration and Physical Fitness in Prehypertensive and Hypertensive Older Adult Women
Source: Front Physiol. 2021 Mar 10;12:566023. doi: 10.3389/fphys.2021.566023 (PMC8006435; doi:10.3389/fphys.2021.566023)
Supplement: Supplementary file 1 [file Table_1.docx]

| Table A – | Characteristics regarding the weekly frequency of ingestion of item 1 (raw salad), item 2 (cooked vegetables), item 3 (fresh fruits), item 4 (beans), and item 5 (milk or yogurt) of the Consumption Markers Form Of the Ministry of Health, at baseline and post 12 weeks of multicomponent physical training |
| --- | --- |

|  | **Item 1** | | **Item 2** | | **Item 3** | | **Item 4** | | **Item 5** | |
| --- | --- | --- | --- | --- | --- | --- | --- | --- | --- | --- |
| **Frequency** | **Baseline** | **Post** | **Baseline** | **Post** | **Baseline** | **Post** | **Baseline** | **Post** | **Baseline** | **Post** |
|  |  |  |  |  |  |  |  |  |  |  |
| **0** | 3 (5,77) | 0 (0) | 1 (1,92) | 0 (0) | 2 (3,85) | 3 (5,77) | 1 (1,92) | 1 (1,92) | 7 (13,46) | 8 (15,38) |
| **1** | 2 (3,85) | 2 (3,85) | 6 (11,54) | 1 (1,92) | 2 (3,85) | 0 (0) | 5 (9,62) | 4 (7,69) | 2 (3,85) | 1 (1,92) |
| **2** | 2 (3,85) | 5 (9,62) | 7 (13,46) | 10 (19,23) | 4 (7,69) | 5 (9,62) | 7 (13,46) | 5 (9,62) | 4 (7,69) | 2 (3,85) |
| **3** | 6 (11,54) | 5 (9,62) | 9 (17,31) | 6 (11,54) | 10 (19,23) | 6 (11,54) | 5 (9,62) | 5 (9,62) | 4 (7,69) | 6 (11,54) |
| **4** | 5 (9,62) | 5 (9,62) | 4 (7,69) | 7 (13,46) | 4 (7,69) | 6 (11,54) | 5 (9,62) | 5 (9,62) | 2 (3,85) | 3 (5,77) |
| **5** | 5 (9,62) | 7 (13,46) | 7 (13,46) | 5 (9,62) | 7 (13,46) | 7 (13,46) | 2 (3,85) | 5 (9,62) | 4 (7,69) | 4 (7,69) |
| **6** | 3 (5,77) | 7 (13,46) | 1 (1,92) | 7 (13,46) | 0 (0) | 7 (13,46) | 4 (7,69) | 5 (9,62) | 1 (1,92) | 8 (15,38) |
| **7** | 26 (50) | 21 (40,38) | 17 (32,69) | 16 (30,77) | 23 (44,23) | 18 (34,62) | 23 (44,23) | 22 (42,31) | 28 (53,85) | 20 (38,46) |
| **p value** | 0,49 | | 0,12 | | 0,10 | | 0,97 | | 0,27 | |
|  |  | |  | |  | |  | |  | |

The data are presented as the number of respondents (%). Fischer's exact test.

| Table B – | Characteristics regarding the weekly frequency of ingestion of item 6 (chips and fried snacks), item 7 (hamburgers and sausages), item 8 (savory cookies), item 9 (sweet cookies, candies, and chocolates), and item 10 (soft drink)^a^ of the Consumption Markers Form Of the Ministry of Health, at baseline and post 12 weeks of multicomponent physical training |
| --- | --- |

|  | **Item 6** | | **Item 7** | | **Item 8** | | **Item 9** | | **Item 10** | |
| --- | --- | --- | --- | --- | --- | --- | --- | --- | --- | --- |
| **Frequency** | **Baseline** | **Post** | **Baseline** | **Post** | **Baseline** | **Post** | **Baseline** | **Post** | **Baseline** | **Post** |
|  |  |  |  |  |  |  |  |  |  |  |
| **0** | 32 (61,54) | 29 (55,77) | 24 (46,15) | 30 (57,69) | 24 (46,15) | 29 (55,77) | 19 (36,54) | 20 (38,46) | 23 (44,23) | 25 (48,08) |
| **1** | 18 (34,62) | 18 (34,62) | 15 (28,85) | 13 (25) | 13 (25) | 9 (17,31) | 13 (25) | 10 (19,23) | 11 (21,15) | 10 (19,23) |
| **2** | 0 (0) | 2 (3,85) | 9 (17,31) | 7 (13,46) | 8 (15,38) | 8 (15,38) | 8 (15,38) | 9 (17,31) | 4 (7,69) | 8 (15,38) |
| **3** | 0 (0) | 0 (0) | 1 (1,92) | 2 (3,85) | 2 (3,85) | 4 (7,69) | 2 (3,85) | 8 (15,38) | 8 (15,38) | 4 (7,69) |
| **4** | 1 (1,92) | 2 (3,85) | 3 (5,77) | 0 (0) | 0 (0) | 0 (0) | 3 (5,77) | 2 (3,85) | 1 (1,92) | 0 (0) |
| **5** | 0 (0) | 1 (1,92) | 0 (0) | 0 (0) | 3 (5,77) | 1 (1,92) | 4 (7,69) | 2 (3,85) | 2 (3,85) | 2 (3,85) |
| **6** | 0 (0) | 0 (0) | 0 (0) | 0 (0) | 0 (0) | 0 (0) | 1 (1,92) | 0 (0) | 1 (1,92) | 2 (3,85) |
| **7** | 1 (1,92) | 0 (0) | 0 (0) | 0 (0) | 2 (3,85) | 1 (1,92) | 2 (3,85) | 1 (1,92) | 2 (3,85) | 1 (1,92) |
| **p value** | 0,61 | | 0,41 | | 0,70 | | 0,52 | | 0,76 | |
|  |  | |  | |  | |  | |  | |

The data are presented as the number of respondents (%). Fischer's exact test. ^a^did not consider diet or light.

| Table C – | Characteristics regarding the practice of physical activity, at baseline and post 12 weeks of multicomponent training |
| --- | --- |

|  | **Baseline** | **Post** | **∆%** |  |
| --- | --- | --- | --- | --- |
|  |  |  |  |  |
| **Walking (min/week)** | 124,7 (119,2) | 138 (117) | 10,7 |  |
| **Moderate PA (min/week)** | 195,8 (256,4) | 242,7 (199,7)* | 24 |  |
| **Vigorous PA (min/week)** | 23,1 (58) | 42,1 (55,7)* | 82,3 |  |
| **ST on the week/day (min)** | 208,5 (128,5) | 181,5 (105,9) | -13 |  |
| **ST on the weekend/day (min)** | 269,2 (172,4) | 239,3 (127,3) | -11,2 |  |
|  |  |  |  |  |
| Data are reported as means (SD). PA: physical activity; ST: sitting time. *p < 0,05 compared with baseline (same group). Linear mixed-effects models. | | | | |
